# Intravenous thrombolytic therapy for acute ischemic stroke in Hubei, China: a survey of thrombolysis rate and barriers

**DOI:** 10.1186/s12883-019-1418-z

**Published:** 2019-08-22

**Authors:** Yanfeng Zhou, Shijiao Yan, Xingyue Song, Yanhong Gong, Wenzhen Li, Mengdie Wang, Xiaoxv Yin, Bo Hu, Zuxun Lu

**Affiliations:** 1 Department of Social Medicine and Health Management, School of Public Health Tongji Medical College, Huazhong University of Science and Technology, Wuhan, 430030 People’s Republic of China; 2 School of Public Health, Hainan Medical University, Haikou, 571199 People’s Republic of China; 3 Key Laboratory of Emergency and Trauma of Ministry of Education, Hainan Medical University, Haikou, 571199 People’s Republic of China; 4 Department of Neurology, Union Hospital Tongji Medical College, Huazhong University of Science and Technology, Wuhan, 430030 People’s Republic of China

**Keywords:** Thrombolysis, Stroke, Neurologists, Emergency medical services, China, Risk factor

## Abstract

**Background:**

Rates of thrombolysis in most countries are well below best practice benchmarks. We aimed to investigate thrombolysis utilization and its associated factors in acute ischemic stroke (AIS) patients in Hubei province, China, to assess neurologists’ experiences of the treatment, and to identify barriers against the treatment from perspective of AIS patients and neurologists.

**Methods:**

Survey of 2096 AIS patients and 709 neurologists from 66 hospitals was conducted in Hubei province between 2014 and 2015. A multivariable logistic regression model was utilized to identify the factors associated with thrombolysis utilization and neurologists’ experiences with thrombolysis.

**Results:**

Of the 2096 AIS patients, only 3.8% received thrombolysis. Of the 709 neurologists, 66.0% reported using thrombolysis for AIS patients. The main reasons for not using thrombolysis were late arrival of patients, fear of the risk of complications of thrombolysis, and light or quickly recovered stroke symptoms. The behavior and clinical characteristics of patients, including early admission to hospital (odds ratio [OR] = 5.81, 95% confidence intervals [CI] 3.31–10.20), using emergency medical services to be hospitalized (OR = 3.36, 95% CI 2.00–5.62), stroke history (OR = 0.53, 95% CI 0.28–0.99), and National Institute of Health Stroke Scale score < 4 (OR = 0.46, 95% CI 0.27–0.77) were shown to significantly affect the thrombolysis utilization in the multivariate model. In addition, hospital grade (OR = 2.84, 95% CI 1.84–4.37), education level (OR = 2.49, 95% CI 1.09–5.73), and working years (OR = 1.88, 95% CI 1.18–3.00) were strongly associated with neurologists’ experiences of thrombolysis.

**Conclusions:**

A very low proportion of AIS patients received thrombolysis in Hubei province, China. Considerable education programs and interventions were required regarding knowledge of stroke treatment for clinicians and proper behavior after stroke for AIS patients and their families.

**Electronic supplementary material:**

The online version of this article (10.1186/s12883-019-1418-z) contains supplementary material, which is available to authorized users.

## Background

In China, there are approximately 2.5 million new stroke cases each year and the most common subtype of stroke is acute ischemic stroke (AIS) [1, 2]. Currently, intravenous thrombolysis with Recombinant Tissue-type Plasminogen Activator (rt-PA) is the only effective treatment for AIS [3, 4]. Although rt-PA has been licensed in the United States, Canada, European Union and some other countries for more than 10 years, rates of thrombolysis in the majority of these countries are well below best practice benchmarks [5]. The Paul Coverdell National Acute Stroke Registry showed that rt-PA was administered to 3.0–8.5% of ischemic stroke patients [6]. The Registry of the Canadian Stroke Network found that the rate of thrombolysis was 8.1–10.2% [7, 8]. The Arbeitsgemeinschaft Deutscher Schlaganfall-Register reported that 3.0–4.0% of AIS patients received rt-PA treatment during the study period [9, 10]. The date from the Stroke Knowledge Network Netherlands dataset showed that mean national thrombolysis rate was 6.4–14.6% between 2005 and 2012 [11]. Cocho et al. [12] conducted a study in Spain and found that only 1 to 7% of AIS patients received the thrombolytic therapy. In China, rt-PA treatment was approved by the State Food and Drug Administration (SFDA) in treating AIS patients if they fulfilled eligibility criteria (see Additional file 1: Table S1) [13]. Combined with the data of China National Stroke Registry (from September 2007 to August 2008) and China Quality Evaluation of Stroke Care and Treatment project (from July 2007 to November 2007), only approximately 1.2–1.9% of AIS patients are treated with rt-PA [14]. Another studies carried out in 7 cities of China in 2006 have showed that 2.7% of AIS patients received thrombolytic therapy [15], whose rates are lower than developed countries. Thus, it is of great significance to explore reasons for low thrombolysis rate in developing countries.

There are several explanations for the low thrombolysis rate. Such as the strict contraindications to thrombolysis, lacking of early neurologic attention, being limited only to rapid emergency care, and having no access to imaging examinations [5, 12]. In previous studies, the most common reasons for not using treatment are late arrival of patients and uncertainty thrombolysis prescribed by physicians. Controlled multicenter studies have demonstrated that thrombolysis treatment must be started within 4.5 h of stroke onset [16, 17]. However, even in developed countries, only approximately 20.0 to 35.8% of patients can meet the treatment window [6, 18, 19]. Additionally, physicians’ decision-making may be different due to the uncertainty of the risks vs. benefits of thrombolysis. Previous studies had shown that the awareness of thrombolysis, fear of risk of bleeding, and doubt of impact of thrombolysis may affect physicians’ decision-making [20, 21].

Nonetheless, most of those studies are conducted in developed countries, and just focused on single viewpoints of patients or neurologists. Studies based on the cognition and behavior of patients and neurologists in China have been limited. As core members of a stroke team, AIS patients and neurologists play important roles in the utilization of thrombolysis. Therefore, we develop this study to describe the use of rt-PA for AIS patients in Hubei province, China, to assess neurologists’ experiences of the treatment, to identify barriers against the treatment from perspective of AIS patients and neurologists, and to suggest improvements to overcome these barriers.

## Methods

### Settings and patients

This cross-sectional study is an investigation of rate of rt-PA use in AIS patients and the reasons for not giving thrombolysis. Details of the study design have been reviewed previously [22, 23]. In brief, we enrolled all patients admitted for AIS, which was defined by the International Classification of Disease, 10th version (ICD-10) code I63 from 66 hospitals in 13 major cities across Hubei province in China. The inclusion criteria for AIS patients were as follows: (1) Patients were hospitalized between November 1, 2014 and January 31, 2015 and were diagnosed with AIS at age 18 years or older; (2) AIS was clinically diagnosed by neuro-imaging (CT or MRI). (3) Stroke survivors. Additionally, neurologists from 66 hospitals were also investigated if they had been in charge of treatment of acute stroke and had been practicing for more than 1 year.

### Data collection

On the basis of reviewing literature and policy analysis, questionnaires were designed for AIS patients and neurologists. All eligible patients and neurologists were interviewed by face-to-face with trained graduate students. When it was not possible to speak with a patient (because of aphasia, low level of consciousness or language differences), the caretakers or family members of the patient were interviewed. Questionnaire for AIS patients documented age, gender, educational level, financial status, residence, medical insurance, living condition, pre-hospital delay, admission form, diseases history, stroke severity, use of thrombolysis, satisfaction with treatment, and the reasons for not using thrombolysis. Questionnaire for neurologists documented age, gender, educational level, job title, hospital grade, working duration in hospital, experiences of thrombolysis, efficacy of rt-PA, and barriers to the utilization of thrombolysis.

### Explanatory variables

Most of the variables were self-explanatory, but a few needed explanation. Based on the overall income level of Hubei province, we divided per capital monthly household income into 4 categories (≤1000, 1000–3000, 3000–5000, and > 5000 Chinese Yuan RMB). We further defined pre-hospital delay measure into 3 or more hours from symptom onset until the earliest documented arrival in emergency departments of hospital. Admission form was divided into “by emergency medical services (EMS)” and “not EMS”. For patients’ medical history, coronary artery disease included both myocardial infarction and angina. Stroke severity was determined use a retrospectively derived National Institutes of Health Stroke Scale (NIHSS) score. NIHSS score < 4 is defined as a light stroke [24]. Satisfaction with treatment was divided into “satisfied”, “general”, and “not satisfied”. For job title of neurologists, four major categories: resident, attending physician, associate chief physician, and chief physician were classified in terms of their competence levels and years of service. With respect to hospital grade, grade-III hospital and grade-II hospital were rated according to their capacity and functions. Specifically, grade-III hospitals round up the list as comprehensive or general hospital at the city, provincial or national level with a bed capacity exceeding 500. Grade-II hospitals tend to be affiliated with a medium size city, county or district and contain more than 100 beds, but less than 500. To describe the experience of thrombolysis, a question “Have you ever done thrombolytic therapy for AIS patients” was provided to each neurologist. The effect of physician-patient relationship on thrombolytic therapy was defined as “negative impact”, “positive impact’, or “no impact” by answering a question “How will the current situation of the worsening physician-patient relationship affect thrombolytic therapy”. As reported in the medical literatures, efficacy of rt-PA was classified as “very convincing”, “somewhat convincing”, or “not convincing”.

### Statistical analysis

Double entry of quantitative data was performed by Epidata 3.0, and the data was analyzed by SPSS 18.0. Descriptive analysis was carried out to preliminarily analyze the data. Chi-square test was used to compare the utilization rate of thrombolysis between groups. Multivariate logistic regression analysis was undertaken to identify the predictors associated with thrombolysis utilization. Adjusted odds ratios (ORs) and 95% confidence intervals (CIs) for each variable were calculated. For all comparisons, differences were tested using two-tailed tests and *p*-values less than 0.05 were considered statistically significant.

## Results

### Characteristics of study subjects

A total of 2176 AIS patients and 719 neurologists completed the questionnaires, of which 80 patients’ questionnaires and 10 neurologists’ questionnaires were excluded because of missing data or other reasons. The overall response rates for patients and neurologists were 96.3% and 98.6% respectively. Tables 1 and 2 showed the demographic characteristics of the patients and neurologists. Of the 2096 patients, the average age was 65.5 (Standard deviation = 12.0) years, with 62.8% men. Of the 709 neurologists, the average age was 35.6 (Standard deviation =7.6) years, with 56.8% men.

**Table 1:** Characteristics of AIS patients

Factors	Total (%)	Used rt-PA		*χ* ^*2*^	*P*
		Yes	No		
Age, year					
< 40	40 (1.9)	5 (12.5)	35 (87.5)	9.766	0.008
41–65	1008 (48.1)	41 (4.1)	967 (95.9)		
> 65	1048 (50.0)	33 (3.1)	1015 (96.9)		
Gender					
Male	1316 (62.8)	65 (4.9)	1251 (95.1)	13.350	0.000
Female	780 (37.2)	14 (1.8)	766 (98.2)		
Ethnicity					
Han nationality	2052 (97.9)	78 (3.8)	1974 (96.2)	0.277	0.598
Non-Han nationality	44 (2.1)	1 (2.3)	43 (97.7)		
Education					
Education ≤6 years^a^	810 (38.6)	17 (2.1)	793 (97.9)	10.155	0.001
Education > 6 years	1286 (61.4)	62 (4.8)	1224 (95.2)		
The per capital monthly household income (yuan)					
> 5000	298 (14.2)	9 (3.0)	289 (7.0)	0.904	0.824
3000–5000	1016 (48.5)	40 (3.9)	976 (96.1)		
1000–3000	656 (31.3)	24 (3.7)	632 (96.3)		
≤ 1000	126 (6.0)	6 (4.8)	120 (95.2)		
Medical insurance					
Yes	2030 (96.9)	78 (3.8)	1952 (96.2)	0.954	0.329
No	66 (3.1)	1 (1.5)	65 (98.5)		
History of hypertension					
Yes	1457 (69.5)	52 (3.6)	1405 (96.4)	0.528	0.468
No	639 (30.5)	27 (4.2)	612 (95.8)		
History of diabetes^b^					
Yes	427 (20.4)	17 (4.0)	410 (96.0)	0.067	0.796
No	1669 (79.6)	62 (3.7)	1607 (96.3)		
History of hyperlipidemia					
Yes	233 (11.1)	10 (4.3)	223 (95.7)	0.198	0.657
No	1863 (88.9)	69 (3.7)	1794 (96.3)		
History of coronary artery disease^c^					
Yes	218 (10.4)	8 (3.7)	210 (96.3)	0.007	0.935
No	1878 (89.6)	71 (3.8)	1807 (96.2)		
History of cardiac arrhythmia					
Yes	72 (3.4)	2 (2.8)	70 (97.2)	0.202	0.653
No	2024 (96.6)	77 (3.8)	1947 (96.2)		
Previous stroke					
Yes	683 (32.6)	15 (2.2)	668 (97.8)	6.911	0.010
No	1413 (67.4)	64 (4.5)	1349 (95.5)		
Admitted to hospital by EMS					
Yes	323 (15.4)	39 (12.1)	284 (87.9)	72.617	0.000
No	1773 (84.6)	40 (2.3)	1733 (97.7)		
NIHSS					
< 4	1090 (52.0)	26 (2.4)	1064 (97.6)	11.989	0.001
≥ 4	1006 (48.0)	53 (5.3)	953 (94.7)		
Pre-hospital delay					
< 3 h	646 (30.8)	60 (9.3)	586 (90.7)	78.415	0.000
≥ 3 h	1450 (69.2)	19 (1.3)	1431 (98.7)		

*EMS* emergency medical services, *NIHSS* National institutes of health stroke scale

^a^Defined as illiterate or having only finished primary education

^b^Confirmed diagnosis of Type 1 or 2 diabetes mellitus on admission

^c^Coronary artery disease includes prior myocardial infarction, or angina

**Table 2:** Characteristics of neurologists

Factors	Total (%)	Used rt-PA		*χ* ^*2*^	*P*
		Yes	No		
Age					
< 35	363 (51.2)	209 (57.6)	154 (42.4)	23.574	< 0.001
≥ 35	346 (48.8)	259 (74.9)	87 (25.1)		
Gender					
Male	403 (56.8)	281 (69.7)	122 (30.3)	5.755	0.016
Female	306 (43.2)	187 (61.1)	119 (38.9)		
Education					
Bachelor degree or lower	354 (49.9)	212 (59.9)	142 (40.1)	17.945	< 0.001
Master degree	297 (41.9)	206 (69.4)	91 (30.6)		
Doctorate degree	58 (8.2)	50 (86.2)	8 (13.8)		
Job title					
Attending physician or lower	511 (72.1)	319 (62.4)	192 (37.6)	13.97	0.001
Associate chief physician	134 (18.9)	95 (70.9)	39 (29.1)		
Chief physician	64 (9.0)	54 (84.4)	10 (15.6)		
Working duration					
< 8	340 (48.0)	191 (56.2)	149 (43.8)	28.146	< 0.001
≥ 8	369 (52.0)	277 (75.1)	92 (24.9)		
Hospital grade^a^					
Grade III	524 (73.9)	382 (72.9)	142 (27.1)	42.517	< 0.001
Grade II	185 (26.1)	86 (46.5)	99 (53.5)		
Physician-patient relationship tense					
Negative impact	571 (80.5)	368 (64.4)	203 (35.6)	3.182	0.074
Positive or no impact	138 (19.5)	100 (72.5)	38 (27.5)		

^a^Grade-III hospital and grade-II hospital were rated according to their capacity and functions

### The utilization of rt-PA and barriers

Additional file 1: Table S2 showed the utilization of rt-PA in AIS patients and neurologists. Of the responding AIS patients, 3.8% (*n* = 79) used rt-PA treatment. Of the responding AIS patients admitted to hospital in 3 h after the symptom onset, 12.2% used rt-PA. Among patients who used thrombolysis, 67.1% were satisfied with the treatment effect. Notably, patients who were young, who were male, who had higher education experiences, who admitted to hospital by EMS, whose NIHSS score ≥ 4, and who admitted to hospital in 3 h after the symptoms onset had higher rates of thromblysis. However, patients who had a history of stroke had lower rates of thromblysis.

Of the responding neurologists, 66.0% (*n* = 468) reported using thrombolysis for AIS patients, with a mean frequency of 15.6 times. Among 468 neurologists who had experiences of thrombolysis, 73.1% (*n* = 342) were “convinced” of its efficacy, whereas 2.5% (*n* = 12) were “not convinced” of its efficacy, and 68.8% (*n* = 322) experienced serious complications in the process of thrombolysis. Moreover, 80.5% of neurologists believed that current situation of the worsening physician-patient relationship had a negative impact on the development of thrombolysis. Notably, neurologists who aged 35 years or more, who were male, who had higher education level, whose job title was associate chief physician or above, whose working duration was ≥8 years, and who worked in grade III hospital had more experience of thrombolysis.

The main reasons for not using thrombolysis in AIS patients were late arrival of patients (37.5%), fear of the risk of complications of thrombolysis (refusal of patients or their families, 33.2%), and light or quickly recovered stroke symptoms (19.2%). The main reasons for patients who had been admitted to hospital in the time window but not been given thrombolysis were refusal to use thrombolysis by patients or their families (43.7%), light or quickly recovered stroke symptoms (26.6%), or other reasons (9.9%). The main barriers to the utilization of thrombolysis for neurologists were the delay of patient presentation (62.8%), fear of the risk of intracerebral haemorrhage (23.7%), and lack of necessary equipment in hospital (3.7%).

### Factors associated with the utilization and experiences of thrombolysis

Tables 3 and 4 presented the results of logistic regressions on potential risk factors for thrombolysis utilization in AIS patients and experiences of thrombolysis in neurologists. After adjusted by patients’ medical history of hypertension, diabetes, hyperlipidemia, coronary artery disease, and cardiac arrhythmia, factors significantly associated with higher thrombolysis utilization were: patients who were male (OR = 2.56), ones with higher education (OR = 2.08), those admitted to hospital by EMS (OR = 3.36), and patients admitted to hospital in 3 h after the onset of symptoms (OR = 5.81). However, older patients (OR = 0.25), and those having a history of stroke (OR = 0.53), patients with lower NIHSS score (OR = 0.46) were associated with lower rate of thrombolysis. Additionally, multivariate logistic regression analysis showed that factors significantly associated with neurologists’ experiences of thrombolysis were: neurologists who had a doctorate degree (OR = 2.49), neurologists who had longer working duration (OR = 1.88), and neurologists who worked in grade III hospital (OR = 2.84).

**Table 3:** Logistic regression model of potential determinants for thrombolysis use in AIS patients

Factor	*P*	OR(95%CI)^b^
Age		
< 40		1
41–65	0.049	0.31 (0.10–0.99)
> 65	0.025	0.25 (0.08–0.84)
Male	0.004	2.56 (1.35–4.84)
Education > 6 years^a^	0.023	2.08 (1.11–3.90)
The per capital monthly household income (yuan)		
> 5000		1
3000–5000	0.792	1.17 (0.36–3.84)
1000–3000	0.605	1.29 (0.49–3.42)
≤ 1000	0.852	0.91 (0.33–2.48)
No medical insurance	0.184	0.25 (0.03–1.93)
Previous stroke	0.047	0.53 (0.28–0.99)
Admitted to hospital by EMS	0.000	3.36 (2.00–5.62)
NIHSS< 4	0.003	0.46 (0.27–0.77)
Pre-hospital delay time ≤ 3 h	0.000	5.81 (3.31–10.20)

*CI* confidence intervals, *EMS* emergency medical services, *NIHSS* National institutes of health stroke scale, *OR* Odds ratio

^a^Defined as illiterate or having only finished primary education

^b^Adjusted for all other variables plus patients’ medical history of hypertension, diabetes, hyperlipidemia, coronary artery disease, and cardiac arrhythmia

**Table 4:** Logistic regression model of potential determinants for experiences of thrombolysis in neurologists

Factor	*P*	OR(95%CI)
Age < 35	0.148	0.70 (0.43–1.14)
Male	0.051	1.41 (0.99–1.99)
Education		
Bachelor degree or lower		1
Master degree	0.563	1.13 (0.75–1.70)
Doctorate degree	0.031	2.49 (1.09–5.73)
Job title		
Attending physician or lower		1
Associate chief physician	0.281	0.74 (0.43–1.28)
Chief physician	0.304	1.51 (0.69–3.33)
Working duration ≥8	0.008	1.88 (1.18–3.00)
Hospital grade = grade III	0.000	2.84 (1.84–4.37)
Physician-patient relationship (negative)	0.076	0.67 (0.44–1.04)

*OR* Odds ratio, *CI* confidence intervals

## Discussion

This current study showed that the rate of thrombolysis in Hubei province, China was 3.8%, which was far lower than the approximately 8% prevalence of thrombolysis in developed countries [6, 7, 9, 10], but close to the approximately 2% prevalence of thrombolysis in some other early studies in China [13, 15], indicating big gaps and ample opportunities in improving AIS thrombolytic treatment in central China.

In both view of neurologists and AIS patients, the main reasons for not using thrombolysis were late arrival of patients and the fear of the risk of complications. In previous studies, a substantial proportion of patients experienced admission delay [6, 18, 19]. A study conducted in India reported that 29% patients presented within 3 h of onset [25]. Other studies conducted in Korea found that between 26.0 and 43.3% of patients arrived within 3 h of onset [26, 27]. Synthesized the results of China National Stroke Registry, China Quality Evaluation of Stroke Care and Treatment project, and study of seven cities in China, we found that approximately 70–80% of AIS patients presented hospitals after 3 h of stroke onset [13, 15]. This means that, given the annual incidence of AIS in China (approximately 4900 000), more than 3430 000 patients would be ineligible for thrombolysis because of late presentation. The probable reason may be that most stroke patients or families cannot properly recognize the stroke symptoms or ignore the severity of symptoms or lack knowledge of care seeking behavior after stroke onset, especially when patients’ symptoms were light or quickly improved, or patients waiting for the improvement of symptoms.

As expected, a risk of bleeding, particularly intracerebral haemorrhage (ICH), can be life-threatening and was the main risk for thrombolysis, which limited the application of thrombolysis to some extent. Brown et al. [21] revealed that 65% of physicians were reported not likely to use rt-PA because of the risk of ICH. Wang et al. [13] showed that many Chinese physicians still overemphasized the adverse effect of thrombolysis, such as risk of ICH, for the complications may aggravate stroke symptoms and cause a conflict between doctors and patients. Especially in the current complicated medical environment in China, more and more physicians prefer to adopt conservative treatment to reduce the medical conflicts caused by complications of thrombolysis. Such an attitude would have a negative impact on patients, who would probably refuse this treatment. In the present study, 80.0% of neurologists believed that the tension between doctors and patients had a negative impact on the development of thrombolysis. Possible reasons for this tension may be attributed to patients’ general skepticism of the medical establishment, the conflict of progressive and traditional medicinal approaches, or something specific about rt-PA risks. The promotion of doctor-patient communication was one of the important premises and links to guarantee medical safety. Furthermore, the presence of appropriate staff to monitor and manage suspected complications was also essential to the proper implementation of thrombolysis.

Our study showed that the percentage of patients who were treated with thrombolysis was significantly higher in grade III hospitals than in grade II hospitals, and the multivariate logistic regression analysis also showed that neurologists’ experience was associated with hospital grade. Hospital size has been indicated to be an important predictor of quality of care, such as emergency treatment and short-term mortality for many conditions, including stroke [28]. Hospital level was also positively correlated with other characteristics of the hospital, such as teaching status, medical staffs, medical resources, and specialty services, which were important to stroke care [29]. Higher level hospital was equipped with a more professional medical team, more standard quality-improvement infrastructure, and better multidisciplinary collaboration, thus it can provide higher quality service of diagnosis and treatment of stroke. However, due to the lack of technical equipment, medical personnel related experiences, and other related factors such as difficulty manage of complications, the application of advanced treatments were relatively weak in lower level hospital. Similar results can be seen in other studies [30]. Therefore, stepping up training and attaching importance to improvement in practical skills was important for the wide application of thrombolysis in the region. Remote thrombolytic therapy or telemedicine, which used network audio-visual capabilities to make the superior neurologists instant communication to physicians and patients in long distance, helping making disease assessment and finally making clinical decision, has been proposed as a solution to low utilization of thrombolysis in AIS [31]. Chalouhi et al. [32] conducted a study in 1643 telemedicine stroke consultations and found that 82% hospitals within the telemedicine program reported a mean increase of 55% in IV-tPA use, and the proportion of patients transferred to a primary stroke center after teleconsultation decreased from 44 to 19%. The experience of the University of Pittsburgh Medical Center telestroke network showed that the overall rate of IV tPA increased from 2.8 to 6.8% after starting telemedicine [33]. In addition, education level and working duration had an impact on neurologists’ treatment experiences. Generally speaking, higher education level and longer job experiences were associated with both higher degree of mastering the treatment knowledge and more opportunities to develop thrombolytic therapy.

In this analysis, compared to patients who had no history of stroke, patients with prior stroke experiences were less likely to use thrombolysis. A possible explanation was that prior stroke in the past 3 months was one of the contraindications of intravenous thrombolysis with rt-PA for AIS by the Chinese SFDA. Our study also found that patients admitted to hospital by EMS were associated with higher rate of thrombolysis. Using EMS as an admission form was recommended by many literatures and stroke guidelines, for it can not only play a role in the efficient transfer of patients, but also requiring to act as a mobile stroke unit, which will transfer patients to more suitable hospitals and improve the efficiency of hospital emergency treatment in stroke [23]. These findings highlighted the requirements for intervention programs to further increase the public’s awareness of EMS utilization, comprehensive education of awareness of stroke, and appropriate response after stroke.

The major strength of our study is that we explore the experiences and evaluation of thrombolysis, as well as the reasons for not giving thrombolysis in both neurologists and patients, which can offer scientific basis for the efficient emergency treatment of stroke and help to increase intravenous rt-PA use in China. This study also has a few limitations. The main limitation is that as a cross-sectional design of the study, recall bias would be inherent. Additionally, we only interview those stroke patients who survived, which may affect the representativeness and lead to selection bias. Finally, emergency physicians’ attitude towards thrombolysis greatly affects the future implementation of thrombolysis, as this is a key step in the administration of first-line therapies for stroke, which we will do in the future.

## Conclusions

The current study presented an inadequate use of thrombolysis among AIS patients in Hubei province, China. Hospital grade, education level, and working years were strongly associated with neurologists’ experiences of thrombolysis. In addition, the behavior of patients (including early admission to hospital and using EMS to be hospitalized) was shown to significantly affect the rate of thrombolysis use. Therefore, considerable education programs and interventions were required regarding knowledge of stroke treatment for clinicians and proper behavior after stroke for AIS patients and their families to improve the use of intravenous thrombolysis in China. Our study also suggested that remote thrombolytic therapy or telemedicine might be an effective way for the management of acute ischemic stroke.

## Additional file

Additional file 1: **Table S1** Indications, contraindications and relative contraindications of intravenous thrombolysis with rt-PA for acute ischemic stroke within 3 h. **Table S2** Thrombolysis use in AIS patients and neurologists. This additional file shows the utilization of rt-PA in AIS patients and neurologists. (DOC 75 kb)

## Data Availability

The data that support the findings of this study are available from the corresponding author on reasonable request.

## Abbreviations

AIS: Acute ischemic stroke
CI: Confidence interval
EMS: Emergency medical services
ICD-10: International Classification of Disease, 10th version
ICH: Intracerebral haemorrhage
NIHSS: National Institutes of Health Stroke Scale
OR: Odds ratio
rt-PA: Recombinant Tissue-type Plasminogen Activator
WHO: World Health Organization
