# Supplementary material for: Intravenous thrombolytic therapy for acute ischemic stroke in Hubei, China: a survey of thrombolysis rate and barriers
Source: BMC Neurol. 2019 Aug 22;19:202. doi: 10.1186/s12883-019-1418-z (PMC6704516; doi:10.1186/s12883-019-1418-z)
Supplement: Supplementary file 1 — Table S1 Indications, contraindications and relative contraindications of intravenous thrombolysis with rt-PA for acute ischemic stroke within 3 h. Table S2 Thrombolysis use in AIS patients and neurologists. This additional file shows the utilization of rt-PA in AIS patients and neurologists. (DOC 75 kb) [file 12883_2019_1418_MOESM1_ESM.doc]

**Additional file 1: Table S1: Indications, contraindications and relative contraindications of intravenous thrombolysis with rt-PA for acute ischemic stroke within 3 hours**

| Indications (all should be "yes") |
| --- |
| 1. Symptoms of neurological impairment caused by ischemic stroke |
| 2. Symptom onset <3 hours |
| 3. Patients aged ≥18 years |
| 4. Patients or family members agreed and signed informed consent |
| Contraindications (all should be "no") |
| 1. Intracranial hemorrhage |
| 2. Prior intracranial hemorrhage |
| 3. A history of severe head trauma or stroke in the past 3 months |
| 4. Intracranial tumors, giant intracranial aneurysms |
| 5. Intracranial or intraspinal surgery in the past months |
| 6. Active visceral bleeding |
| 7. Dissected aortic arch |
| 8. Arterial puncture in the past 1 week |
| 9. SBP greater than 180 mm Hg, or DBP greater than 100 mm Hg |
| 10. Acute bleeding tendency, including platelet count below 100*109/L or other conditions |
| 11. Received low molecular weight heparin treatment in the past 24 hours |
| 12. Current use of oral anticoagulants and INR of more than 1.7 or PT of more than 15 s |
| 13. Received thrombin inhibitors or Xa inhibitors within 48 hours |
| 14. Blood glucose levels of lower than 2.8 mmol/L (50 mg/L) or higher than 22.22 mmol/L (400 mg/L) |
| 15. One-third MCA territory revealed by CT/MRI |
| Relative Contraindications (should carefully considered and weighed the risks and benefits of thrombolysis) |
| 1. Mild non-disabling stroke |
| 2. Light or quickly recovered stroke symptoms |
| 3. Seizure with stroke onset |
| 4. Extracranial cervical artery |
| 5. Major surgery or serious trauma in the past 2 weeks |
| 6. Gastrointestinal or urinary bleeding in past 3 weeks |
| 7. Pregnant |
| 8. Dementia |
| 9. Severe neurological disability was inherited from previous diseases |
| 10. Unruptured and untreated arteriovenous malformations and small intracranial aneurysms (<10 mm) |
| 11. Intracerebral microbleeds |
| 12. Use of banned substances |
| 13. Stroke mimics |

Abbreviations: CT, computed tomography; DBP, diastolic bold pressure; INR, international normalized ratio; MCA, middle cerebral artery; MRI, magnetic resonance imaging; PT, prothrombin time; SBP, systolic blood pressure.

**Additional file 1: Table S2: Thrombolysis use in AIS patients and neurologists**

| **Classification** | **Factor** | **Category** | **Total** | **%** |
| --- | --- | --- | --- | --- |
| **Patients (2096)** | Using thrombolysis | Yes | 79 | 3.8 |
|  |  | No | 2017 | 96.2 |
|  | Effects of thrombolysis**^§^** | Very good | 12 | 15.2 |
|  |  | Good | 41 | 51.9 |
|  |  | General | 19 | 24.1 |
|  |  | Bad | 5 | 6.3 |
|  |  | Very bad | 2 | 2.5 |
|  | Reasons for not using thrombolysis | Late arrival | 756 | 37.5 |
|  |  | Symptoms were light or quickly recovered | 387 | 19.2 |
|  |  | High cost of thrombolysis | 56 | 2.8 |
|  |  | Fear of the risk of complications | 671 | 33.2 |
|  |  | Old age | 12 | 0.6 |
|  |  | Contraindications | 20 | 1.0 |
|  |  | Other | 115 | 5.7 |
|  | Reasons for not using thrombolysis in time-window | Missed the treatment time | 52 | 9.2 |
|  |  | Symptoms were light or quickly recovered | 151 | 26.6 |
|  |  | High cost of thrombolysis | 42 | 7.4 |
|  |  | Fear of the risk of complications | 248 | 43.7 |
|  |  | Old age | 9 | 1.6 |
|  |  | Contraindications | 9 | 1.6 |
|  |  | Other | 56 | 9.9 |
| **Neurologists (709)** | Experiences of thrombolysis | Yes | 468 | 66.0 |
|  |  | No | 241 | 34.0 |
|  | Effects of thrombolysis | Very convinced | 51 | 10.9 |
|  |  | Convinced | 291 | 62.2 |
|  |  | General | 114 | 24.4 |
|  |  | Unconvinced | 10 | 2.1 |
|  |  | Very unconvinced | 2 | 0.4 |
|  | Experienced serious complications**^＊^** | Yes | 322 | 68.8 |
|  |  | No | 146 | 31.2 |
|  | Main barriers to the utilization of thrombolysis | The delay of patient presentation | 445 | 62.8 |
|  |  | Lack of experiences or knowledge | 16 | 2.3 |
|  |  | Lack of necessary equipment in hospital | 26 | 3.7 |
|  |  | Failed to gain support of leader of hospital | 18 | 2.5 |
|  |  | the fear of the risk of intracerebral haemorrhage | 169 | 23.7 |
|  |  | Refusal of patients | 13 | 1.8 |
|  |  | Other | 22 | 3.1 |

^§^: Patient self-assessment of pain, satisfaction, quality of life, and so on.

^＊^: Serious complications after thrombolysis included intracerebral hemorrhage and reperfusion injury.
